# Efficacy and safety of bridging intravenous thrombolysis before mechanical thrombectomy: A systematic review and meta-analysis

**DOI:** 10.1177/15910199251368728

**Published:** 2025-09-03

**Authors:** Tallal Mushtaq Hashmi, Mushood Ahmed, Hadiah Ashraf, Muhammad Shakir, Ibrahim Ahmad Bhatti, Ahmad Alareed, Faizan Ahmed, Ali Hasan, Raheel Ahmed, Majid Toseef Aized, Shahid Rafiq, Gregg C Fonarow, Ameer E Hassan

**Affiliations:** 1 Department of Neurology, 123683 Rawalpindi Medical University, Rawalpindi, Pakistan; 2 Department of Neurology, 4139 University of Missouri, Columbia, MO, USA; 3 Department of Neurosurgery, 4139 University of Missouri, Columbia, MO, USA; 4 Department of Neurology, 7425 University Hospital Southampton NHS Foundation Trust, Southampton, UK; 5 Department of Internal Medicine, 23498 Jersey Shore University Medical Center, Neptune, NJ, USA; 6 Department of Medicine, 4615 Imperial College London, London, UK; 7 Department of Cardiology, Royal Brompton Hospital, London, UK; 8 Department of Vascular Surgery, 22804 North Alabama Medical Center, Florence, AL, USA; 9 Adjunct Clinical Faculty, Kentucky College of Osteopathic Medicine, Pikeville, KY, USA; 10 Director Stroke and Neurology, 244117 Holy Cross & Frederick Health, Frederick, MD, USA; 11 Ahmanson-UCLA Cardiomyopathy Center, Division of Cardiology, 8783 University of California, Los Angeles, CA, USA; 12 Department of Neurology, 21755 University of Texas Rio Grande Valley, Valley Baptist Medical Center, Harlingen, TX, USA

**Keywords:** Stroke, thrombolytic therapy, intravenous thrombolysis, thrombectomy

## Abstract

**Background:**

The safety and efficacy of intravenous thrombolysis (IVT) before mechanical thrombectomy (MT) in acute ischemic stroke remain uncertain.

**Methods:**

We comprehensively searched PubMed, Embase, and the Cochrane Library from inception to May 30, 2025. Randomized controlled trials comparing IVT before MT versus MT alone in acute ischemic stroke were included. The primary outcome was excellent functional outcome (modified Rankin Scale score 0–1 at 90 days) and good functional outcome (modified Rankin Scale score 0–2). Secondary outcomes included successful recanalization, all-cause death, symptomatic, and any intracranial hemorrhage. Odds ratios (ORs) with 95% confidence intervals (CIs) were pooled using a random-effects model.

**Results:**

Seven randomized controlled trials encompassing 2884 patients (MT + IVT: 1448; MT–IVT: 1436) met the inclusion criteria. The pooled analysis demonstrated comparable results for excellent functional outcome (31.3% vs. 29.5%; OR, 1.08 [95% CI, 0.92–1.28]), good functional outcome (51.2% vs. 48.0%; OR, 1.13 [95% CI, 0.96–1.34]) between the MT + IVT and MT alone groups, respectively. Rates of successful recanalization (OR, 1.24 [95% CI, 0.95–1.62]), all-cause death (OR, 0.98 [95% CI, 0.80–1.19]), symptomatic intracranial hemorrhage (OR, 1.21 [95% CI, 0.87–1.68]), and any intracranial hemorrhage (OR, 1.17 [95% CI, 0.97–1.41]) were also comparable between the two groups. Trial sequential analysis demonstrated insufficient evidence to confirm a 20% relative benefit of bridging therapy compared to MT alone.

**Conclusion:**

In this study-level meta-analysis, IVT followed by endovascular treatment showed comparable safety and efficacy to endovascular treatment alone, with similar outcomes in functional recovery, successful recanalization, all-cause mortality, symptomatic intracranial hemorrhage, and any intracranial hemorrhage.

## Introduction

Acute ischemic stroke (AIS) remains a leading cause of morbidity and mortality globally, with its management continually advancing through innovations in endovascular therapy.^1^ Intravenous thrombolysis (IVT) before mechanical thrombectomy (MT) may help improve distal perfusion and functional recovery in stroke patients.^2,3^ Concurrently, IVT remains a standard component of pre-endovascular protocols, as endorsed by the American Heart Association/American Stroke Association and the European Stroke Organization, both of which support its use before MT in patients with AIS due to anterior circulation large vessel occlusion.^4,5^ Compared to standard medical therapy alone, MT has been shown to improve functional recovery in AIS patients.^6^ However, the potential benefit of IVT before MT remains unclear.

A number of trials have tried to address this question.^2,7–12^ The primary objective of the majority of these trials was to evaluate the noninferiority of endovascular treatment alone in comparison to the combined approach of IVT followed by endovascular therapy. Recently, the findings from BRIDGE-TNK have been published.^2^ The findings from this trial showed that the percentage of patients with functional independence at 90 days was higher with IVT plus MT than with MT alone. This, along with prior randomized controlled trials (RCTs), highlights the need for a more precise estimate of treatment effect, as individual trials have been limited by moderate sample sizes and wide confidence intervals (CIs) around effect estimates.

Previous study-level meta-analyses have been constrained by the limited number of available RCTs, reliance on post hoc analyses of RCTs, and the inclusion of observational data.^13,14^ Therefore, we aim to conduct an updated meta-analysis synthesizing data exclusively from RCTs to provide the most robust and definitive evidence on this topic and inform clinical decision-making. Additionally, we extend the evidence base by performing a certainty assessment and trial sequential analysis (TSA) to evaluate the quality and conclusiveness of the available evidence and to determine whether future studies are likely to impact the overall conclusions.

## Methods

This meta-analysis was conducted in accordance with the PRISMA guidelines^15^ (Table S1). The protocol for this meta-analysis has been registered on PROSPERO to ensure transparency and methodological rigor. As this review did not involve any direct patient data collection or investigation by the authors, ethical approval was not needed.

### Literature search

We conducted a comprehensive search of the Cochrane Central Register of Controlled Trials, PubMed, and Embase databases, as well as registered trial repositories, from their inception to May 30, 2025. The search strategy utilized a combination of targeted keywords and Medical Subject Headings terms, such as “acute ischemic stroke,” “intravenous thrombolysis,” “mechanical thrombectomy,” and “RCT.” Boolean operators (AND, OR) were applied to optimize the search and ensure the inclusion of all relevant studies. Further details regarding the search terms and strategy are provided in Tables S2 to S3.

### Eligibility criteria

The inclusion criteria for this review were as follows: (1) study design: RCTs; (2) population: patients diagnosed with AIS resulting from large vessel occlusion; (3) intervention: MT combined with IVT; (4) comparator: MT alone; and (5) outcome: reporting at least one relevant outcome of interest.

No restriction was imposed on the type of thrombolytic used. Studies were excluded if they were not RCTs, experimental studies, phase I trials, post hoc analysis, or observational studies.

### Study selection and data extraction

Rayyan software was utilized to screen and remove duplicates from the studies identified in the online search. Following duplicates removal, two authors independently carried out title and abstract screening. Afterwards, a comprehensive full-text review was performed by the same authors. In instances of disagreement, a third author facilitated resolution.

Study characteristics, such as authors and study locations, inclusion criteria, total population, time from symptom onset to MT, thrombolytic drug and dosage, sample size, age, sex, comorbidities (diabetes, hypertension, stroke/TIA, or atrial fibrillation), prestroke mRS (modified Rankin Scale), baseline NIHSS (National Institutes of Health Stroke Scale score), and ASPECTS (Alberta Stroke Program Early Computed Tomography Score) scores, were extracted. The primary outcome was good functional outcome (mRS 0–2 at 90 days). The secondary outcomes included excellent functional outcome (mRS 0–1 at 90 days), successful recanalization, symptomatic intracranial hemorrhage (sICH), and any intracranial hemorrhage (any ICH). A list of common variable definitions has been provided in Table S4. Detailed inclusion and exclusion criteria of each trial are presented in Table S5.

### Quality assessment

The risk of bias for all included trials was assessed using the Cochrane Risk of Bias 2.0 (RoB 2.0) tool.^16^ Each included study was assessed for bias and categorized as having low, high, or some concerns. Two independent reviewers evaluated the risk of bias in each study. Any discrepancies were addressed through discussions with a third reviewer.

### Statistical analysis

Statistical analysis was conducted using R version 4.41, using the “meta” package. Odds ratios (ORs) with 95% CIs were calculated as summary statistics, utilizing a random-effects model, with the results depicted in forest plots. Heterogeneity was assessed using the χ² test and Higgins’ *I*² statistic, with the following thresholds: 0–40% (low), 30–60% (moderate), 50–90% (substantial), and 75–100% (considerable) heterogeneity.^17^ Publication bias was assessed through visual inspection of funnel plots; however, statistical tests were not conducted due to the limited number of included studies (fewer than 10). A sensitivity analysis utilizing the leave-one-study method was conducted, sequentially excluding each study from the pooled analysis to evaluate its effect on the overall results. *P* values were two-sided with a significance threshold of <0.05. Subgroup analysis was conducted based on the thrombolytic agent used (alteplase vs. tenecteplase). A *p* value of <0.1 was considered indicative of a statistically significant subgroup effect.^18^

Trial Sequential Analysis is an advanced meta-analytical method that evaluates the cumulative evidence from preceding clinical trials in a sequential manner to determine whether sufficient data have been accrued to reach definitive conclusions. This approach mitigates the risks of type I and type II errors commonly encountered in meta-analyses of RCTs characterized by small sample sizes. Trial Sequential Analysis establishes sequential monitoring boundaries to evaluate whether the observed *p*-value for a given outcome provides robust and reliable evidence of the anticipated effect. Crossing these boundaries indicates that the evidence is adequate to support a conclusive inference. Trial Sequential Analysis computations—including information size estimation and boundary construction—were conducted using the Copenhagen Trial Unit's Centre for Clinical Intervention Research software (version 0.9.5.10 Beta). Trial Sequential Analysis was done for primary favorable outcomes, assuming a 20% relative risk increase, with a significance level of 5% and statistical power of 80%. A relative risk increase of 20% was chosen to represent a reasonable clinical intervention effect based on recent clinical trials.^2,10,12^ The event rate in the control group was estimated based on the control arms included in the meta-analysis. In addition, a sensitivity analysis was conducted using a more conservative assumption of a 15% relative increase, maintaining the same significance level and power, to assess the robustness of the findings.

### Certainty of evidence assessment

The certainty of the evidence was evaluated using the Grading of Recommendations, Assessment, Development, and Evaluation (GRADE) framework.^19^ Based on the GRADE Working Group guidelines, the certainty of the pooled estimates was rated as high, moderate, low, or very low (Table S6).

## Results

### Study selection

**Figure 1.**
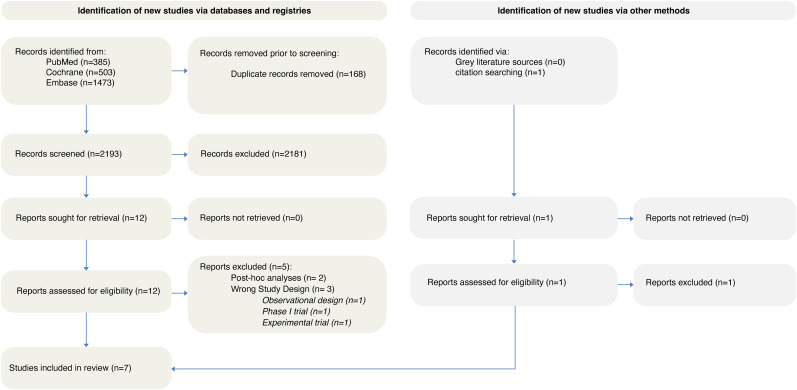
PRISMA flowchart depicting the screening and study selection process.

A total of 2361 records were identified from the databases, including PubMed (385), Cochrane (503), Embase (1473), and citation searching (1). After excluding 168 duplicate entries, 2193 records were screened based on their titles and abstracts. Of these, 2181 records were excluded, and the remaining studies underwent full-text assessment for eligibility. A detailed list of excluded studies at this stage, along with reasons for exclusion, is provided in Table S7. Finally, seven RCTs were deemed eligible and included in the final analysis (Figure 1).

### Characteristics of included studies

A total of seven RCTs were included, conducted across China, Japan, New Zealand, Vietnam, Australia, Europe, and Canada.^2,7–12^ These studies involved a combined total of 2884 participants, with 1448 patients in the MT plus IVT group and 1436 in the MT-only group. The majority of trials employed alteplase as the thrombolytic agent. Tenecteplase was used exclusively in the BRIDGE-TNK trial,^2^ whereas in the DIRECT-SAFE trial,^9^ only 17% of patients received tenecteplase, with the remaining patients treated with alteplase. The median follow-up period across studies was 90 days. The mean age was 69.9 years in the MT + IVT group and 70.4 years in the MT-only group. Most trials predominantly included patients with anterior circulation strokes. However, the DIRECT-SAFE^9^ trial enrolled 19 patients with basilar artery occlusion, and the BRIDGE-TNK^2^ trial included 49 patients with vertebrobasilar artery occlusion. Prevalence rates of medical comorbidities, along with baseline characteristics such as NIHSS, ASPECTS, and mRS scores, are summarized in Table 1. A low risk of bias was observed in all RCTs (Figure S1).

**Table 1.** Baseline characteristics of the included studies and participants.

Trial			DIRECT-MT 2020	SKIP 2021	DEVT 2021	MR CLEAN–NO IV 2021	DIRECT-SAFE 2022	SWIFT DIRECT 2022	BRIDGE TNK 2025
Study location			China	Japan	China	Multinational^†^	Multinational^‡^	Multinational^§^	China
IVT drug			Alteplase	Alteplase	Alteplase	Alteplase	Alteplase / Tenecteplase	Alteplase	Tenecteplase
IVT dose			0.9 mg/ kg	0.6 mg/ kg	0.9 mg/ kg	0.9 mg/ kg	0.9 mg/ kg	0.9 mg/ kg	0.25 mg/ kg
Median follow-up			90 days	90 days	90 days	90 days	90 days	90 days	90 days
Location of occlusion			ICA, MCA (M1, M2)	ICA, MCA (M1, M2)	ICA, MCA (M1, M2)	ICA, MCA (M1, M2)	ICA, MCA (M1, M2), BA	ICA, MCA (M1, M2)	ICA, MCA (M1, M2), VBA
Successful recanalization			eTICI score ≥ 2b	eTICI 2b/2C/3	eTICI 2b/2C/3	eTICI 2b/2C/3	mTICI 2b/2C/3	eTICI 2b50	eTICI 2b/2C/3
Sample size		MT + IVT	329	103	118	266	147	207	278
		MT–IVT	327	101	116	273	146	201	272
Time interval between stroke onset to MT (mins)^¶^		MT + IVT	213 ± 69.2	158 ± 60.3	210 (179–255)	135 (106–185)	193 ± 74.5	NR	192.0 ± 68.4
		MT–IVT	198 ± 62.8	149 ± 64.6	200 (155–247)	130 (103–180)	165 ± 60	NR	187.5 ± 75
Age (years)^¶^		MT + IVT	68.7 ± 11.2	74.3 ± 9.8	69.3 ± 13.5	69 ± 11.9	69.3 ± 14.2	72.7 ± 11.9	69 ± 12.7
		MT–IVT	68.7 ± 11.2	73.7 ± 9.8	69 ± 12.8	71.3 ± 13.4	69.7 ± 12.7	72.7 ± 12.7	69.7 ± 9.7
Male (%)		MT + IVT	55	70	55.9	54.1	60	50	57.9
		MT–IVT	57.8	55	56.9	59	53	48	58.5
Past medical history:									
	Diabetes (%)	MT + IVT	19.8	17	17	18.8	NR	NR	20.5
		MT–IVT	18	16	21.6	14.7	NR	NR	21.3
	Hypertension (%)	MT + IVT	61.1	59	62.7	52.5	61	57	61.5
		MT–IVT	59	60	59.5	44.3	59	60	65.4
	Atrial fibrillation (%)	MT + IVT	45.3	62	52.5	23.7	23	11	36.3
		MT–IVT	46.5	56	53.5	31.5	32	8	35.3
	Stroke/TIA (%)	MT + IVT	14.3	14	16.1	16.5	12	10	18.7
		MT–IVT	13.1	12	12.1	17.2	18	10	18.4
Prestroke mRS score of 1 (%)		MT + IVT	7.3	6	9.3	18.4	NR	13	4
		MT–IVT	8.3	11	5.2	18.8	NR	17	3.3
Baseline NIHSS score^¶^		MT + IVT	17.7 ± 6	17 ± 7.5	16.3 ± 5.3	15.3 ± 7.5	15 ± 7 .5	16.3 ± 6	16 ± 6
		MT–IVT	16.7 ± 6.7	20 ± 11.3	16 ± 6	15.3 ± 7.5	15.3 ± 6.7	16.7 ± 5.2	16 ± 6
Baseline ASPECTS score^¶^		MT + IVT	8.7 ± 2.2	7.7 ± 2.3	8 ± 1.5	9 ± 1.5	9.7 ± 0.8	8 ± 1.5	7.7 ± 2.2
		MT–IVT	8.7 ± 2.2	7.3 ± 2.3	8 ± 1.5	9 ± 1.5	9.7 ± 0.8	8 ± 1.5	7.7 ± 2.2

DIRECT-MT: direct intraarterial thrombectomy in order to revascularize acute ischemic stroke patients with large vessel occlusion efficiently in Chinese Tertiary Hospitals; SKIP: direct mechanical thrombectomy in acute LVO (large vessel occlusion) stroke; DEVT: direct endovascular thrombectomy vs combined IVT and endovascular thrombectomy for patients with acute large vessel occlusion in the anterior circulation; MR CLEAN–NO IV: multicenter randomized clinical trial of endovascular treatment for acute ischemic stroke in the Netherlands; DIRECT-SAFE: a randomized controlled trial of DIRECT endovascular clot retrieval versus standard bridging thrombolysis with endovascular clot retrieval; SWIFT DIRECT: solitaire with the intention for thrombectomy plus intravenous t-PA versus DIRECT solitaire stent-retriever thrombectomy; BRIDGE TNK: randomized trial of thrombectomy with versus without recombinant human tenecteplase (TNK) tissue plasminogen activator in stroke; IVT: intravenous thrombolysis; MT: mechanical thrombectomy; ICA: internal carotid artery; MCA: middle cerebral artery; BA: basilar artery; VBA: vertebrobasilar artery; M1: first segment of the middle cerebral artery; M2: second segment of the middle cerebral artery; eTICI: expanded thrombolysis in cerebral infarction; mTICI: modified thrombolysis in cerebral infarction; mRS: modified Rankin Scale; NIHSS: National Institutes of Health Stroke Scale; ASPECTS: Alberta Stroke Program Early CT Score; TIA: transient ischemic attack; NR: not reported.

¶ Continuous data are reported as mean ± SD or median (IQR).

† Netherlands, Belgium, and France.

‡ New Zealand, China, Vietnam, and Australia.

§ Europe and Canada.

### Primary outcome

#### Good functional outcome

**Figure 2.**
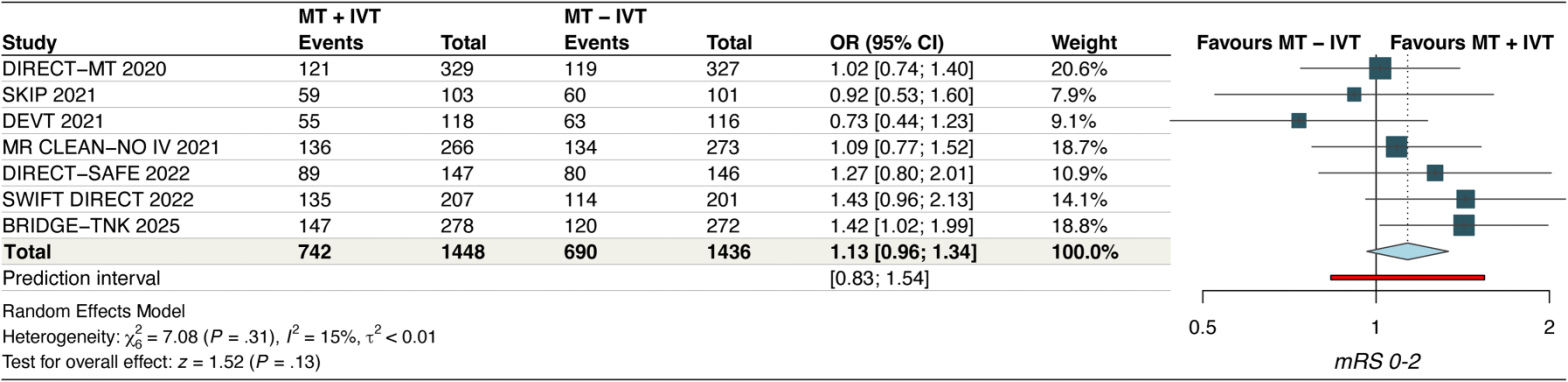
Forest plot for good functional outcome. The pooled odds ratio was 1.13 [95% CI: 0.96–1.34], indicating no statistically significant difference between MT + IVT and MT alone (*p* = 0.13). Heterogeneity was low (*I*² = 15%).

The pooled analysis from seven RCTs demonstrated comparable results for good functional outcome between MT + IVT and MT–IVT (OR, 1.13 [95% CI, 0.96–1.34]; *p* = 0.13, *I*^2^ = 15%, high certainty) (Figure 2). Upon excluding the DEVT RCT, the *I*^2^ decreased to 0% and the finding became statistically significant (OR = 1.19; [95% CI, 1.02–1.39]) (Figure S2). Subgroup analysis based on type of thrombolytic agent revealed no significant subgroup interactions (P*interaction*, 0.13) (Figure S3).

### Secondary outcomes

#### Excellent functional outcome

**Figure 3.**
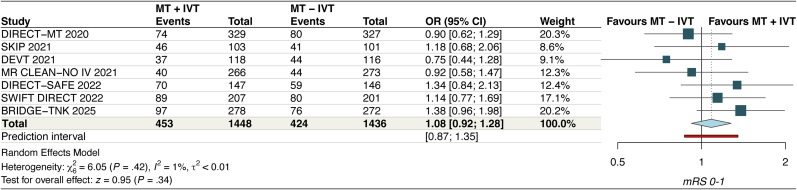
Forest plot for excellent functional outcome. The pooled odds ratio was 1.08 [95% CI: 0.92–1.28], suggesting comparable outcomes between treatment strategies (*p* = 0.34). Heterogeneity was low (*I*² = 1%).

The pooled analysis from seven RCTs demonstrated comparable results for excellent functional outcome between MT + IVT and MT–IVT (OR, 1.08 [95% CI, 0.92–1.28]; *p* = 0.34, *I*^2^ = 1%, high certainty) (Figure 3). Leave-one-out sensitivity analysis did not reveal any significant findings (Figure S4). Subgroup analysis of six RCTs comparing alteplase and tenecteplase revealed a statistically significant subgroup interaction (P*interaction*, 0.09) (Figure S5).

#### Successful recanalization

**Figure 4.**
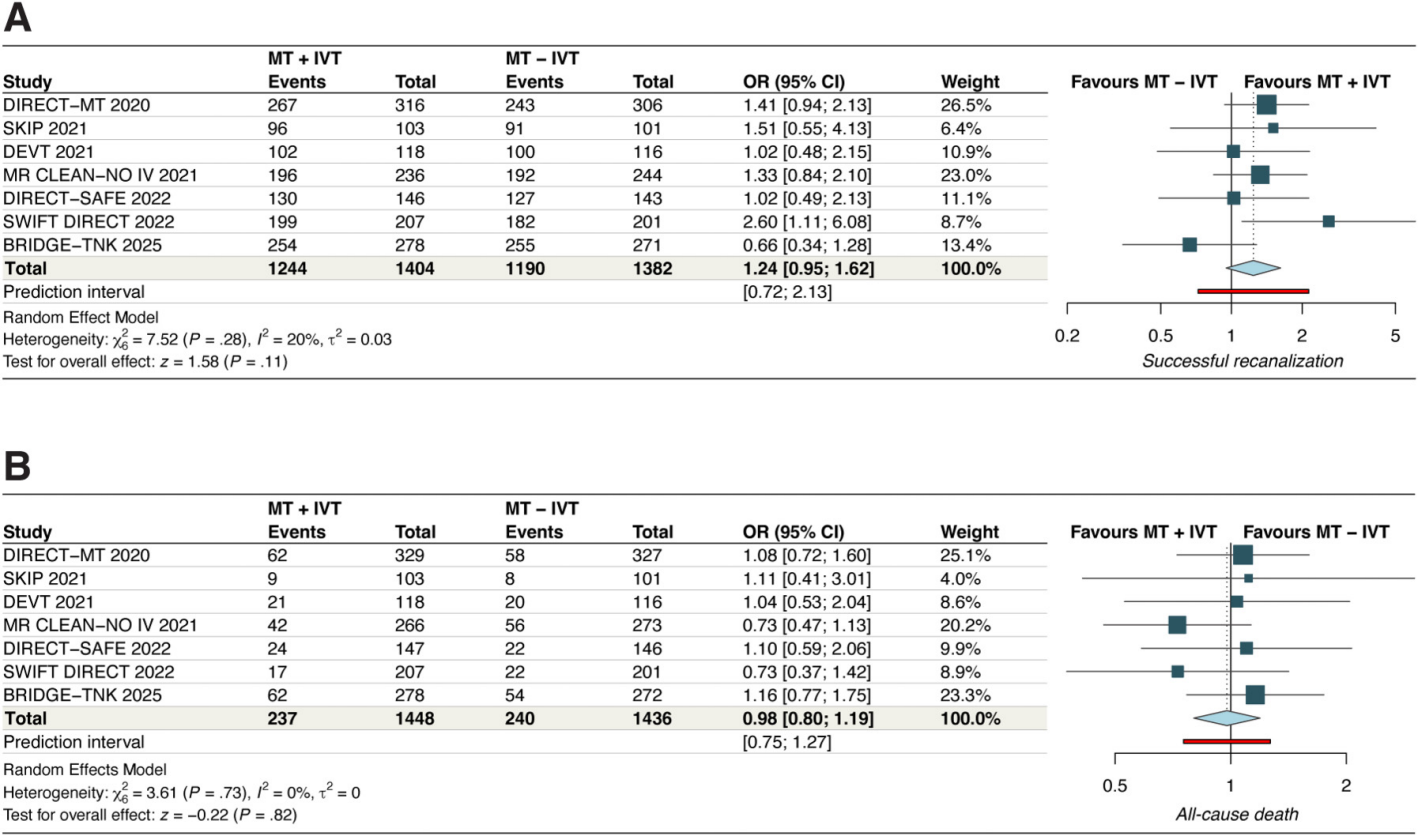
Forest plots for (A) successful recanalization and (B) all-cause death. For successful recanalization, the pooled odds ratio was 1.24 [95% CI: 0.95–1.62], with low heterogeneity (*I*² = 20%), indicating no significant difference between groups. For all-cause death, the pooled odds ratio was 0.98 [95% CI: 0.80–1.19], with no heterogeneity (*I*² = 0%), suggesting comparable mortality rates across treatment arms.

Our pooled analysis from seven studies demonstrated that the odds of successful recanalization are comparable in both groups (OR, 1.24 [95% CI, 0.95–1.62]; *p* = 0.11, *I*^2^ = 20%, moderate certainty) (Figure 4A). A leave-one-out sensitivity analysis by excluding the SWIFT DIRECT and BRIDGE TNK studies reduced the *I*^2^ to 0% (Figure S6). Exclusion of BRIDGE TNK also makes the finding statistically significant (OR = 1.36; [95% CI, 1.07–1.74]) (Figure S6). Significant subgroup interactions were also noted based on thrombolytic agent used (P*interaction*, 0.04) (Figure S7).

#### All-cause death

The pooled analysis of seven RCTs demonstrated comparable results between the two groups (OR, 0.98 [95% CI, 0.80–1.19]; *p* = 0.82, *I*^2^ = 0%, moderate certainty) (Figure 4B). A leave-one-out sensitivity analysis did not reveal any significant findings (Figure S8). No significant subgroup interactions were noted based on thrombolytic agent used (P*intercation*, 0.31) (Figure S9).

#### Symptomatic ICH

**Figure 5.**
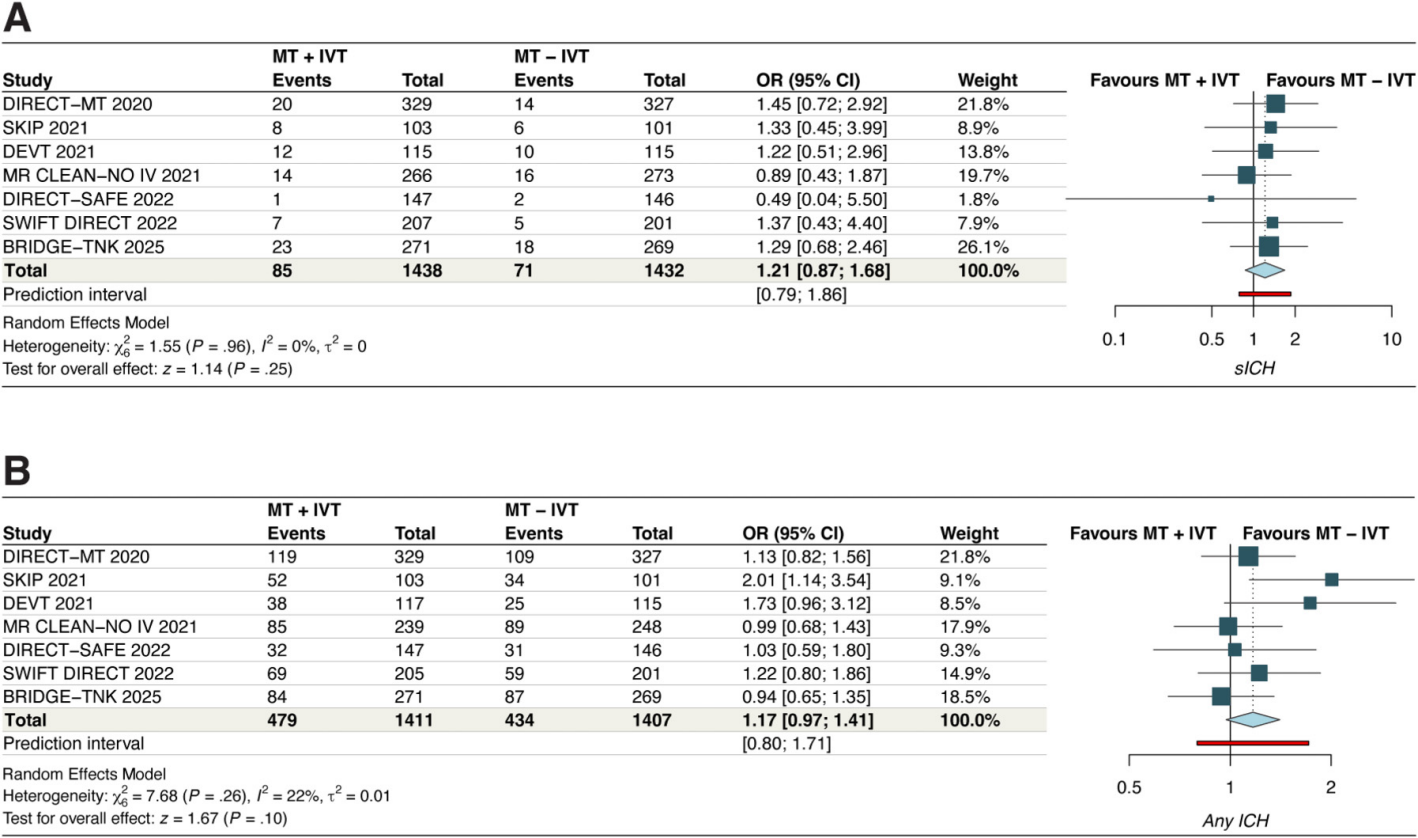
Forest plots for (A) symptomatic intracranial hemorrhage and (B) any intracranial hemorrhage. For symptomatic intracranial hemorrhage, the pooled odds ratio was 1.21 [95% CI: 0.87–1.68], with no heterogeneity (*I*² = 0%, *p* = 0.25), indicating no significant increase in risk with IVT pretreatment. For any intracranial hemorrhage, the pooled odds ratio was 1.17 [95% CI: 0.97–1.41], with low heterogeneity (*I*² = 22%, *p* = 0.10), suggesting a nonsignificant trend toward increased risk in the IVT group.

The analysis demonstrated a nonstatistically significant trend toward a higher incidence of sICH with IVT + MT compared to MT alone (OR, 1.21 [95% CI, 0.87–1.68]; *p* = 0.25, *I*^2^ = 0%, moderate certainty) (Figure 5A). Leave-one-out sensitivity analysis did not reveal any significant findings (Figure S10). No significant subgroup interactions were noted based on thrombolytic agent used (P*intercation*, 0.86) (Figure S11).

#### Any ICH

The pooled analysis demonstrated a nonstatistically significant trend toward a higher incidence of any ICH with IVT + MT compared to MT alone (OR, 1.17 [95% CI, 0.97–1.41]; *p* = 0.1, *I*^2^ = 22%, moderate certainty) (Figure 5B). Leave-one-out sensitivity analysis by excluding the SKIP 2021 study reduced the *I*^2^ to 0% (Figure S12). No significant subgroup interactions were noted based on thrombolytic agent used (P*intercation*, 0.17) (Figure S13).

Publication bias for each outcome was evaluated by visually inspecting funnel plot asymmetry. No notable asymmetry was observed across the funnel plots (Figure S14).

### Results of TSA

**Figure 6.**
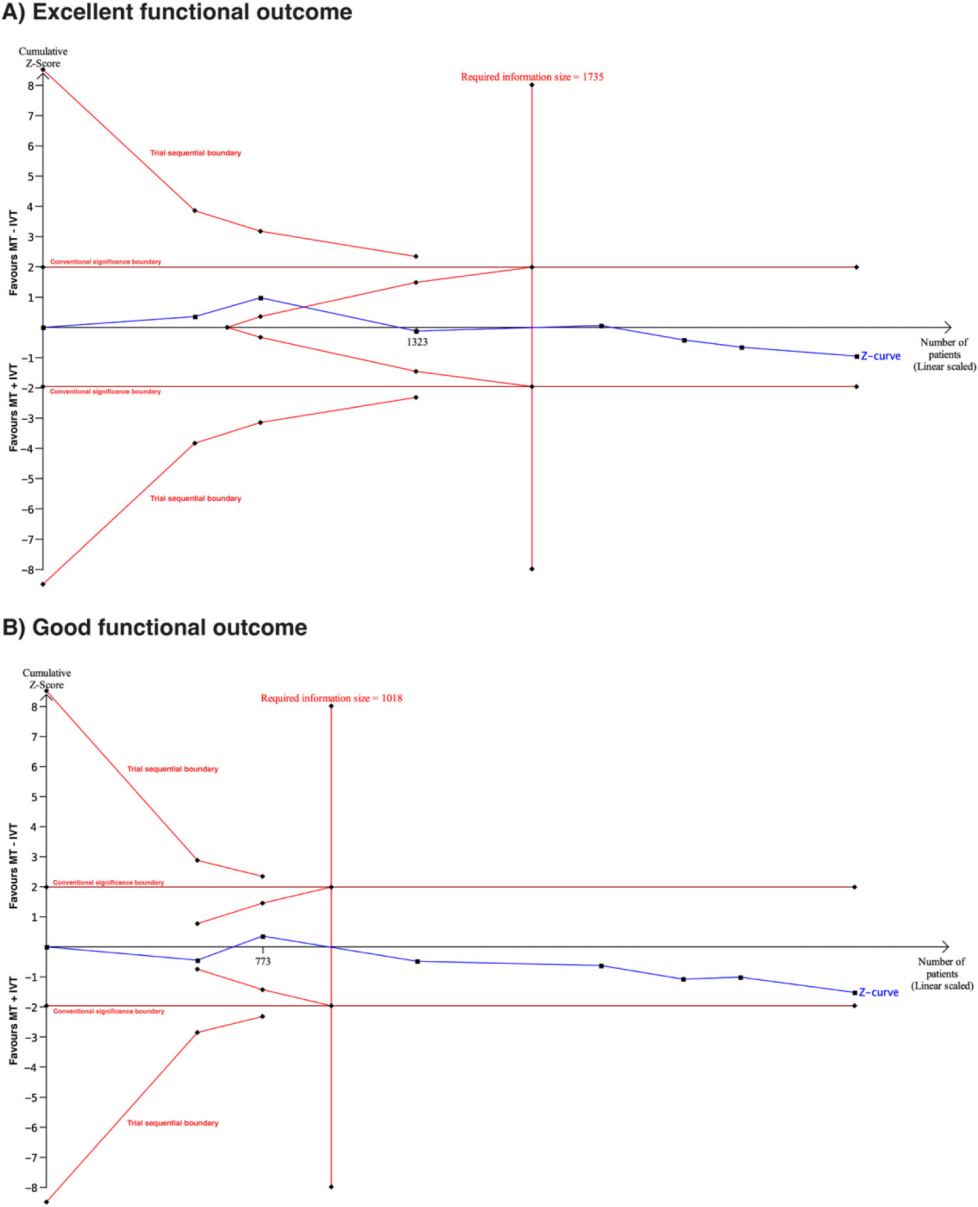
(A) Trial sequential analysis of seven randomized trials assessing excellent functional outcomes (mRS 0–1) with α = 5%, power = 80%, and a 20% relative risk increase. The cumulative Z-score failed to cross the conventional significance boundary as well as trial monitoring boundaries, indicating insufficient evidence to confirm a 20% relative risk increase for MT + IVT over MT–IVT for mRS 0–1. (B) Trial sequential analysis of seven randomized trials assessing good functional outcomes (mRS 0–2) with α = 5%, power = 80%, and 20% relative risk increase. The cumulative Z-score failed to cross the conventional significance boundary as well as trial monitoring boundaries, indicating insufficient evidence to confirm a 20% relative risk increase for MT + IVT over MT–IVT for mRS 0–2.

For favorable functional outcomes (mRS 0–1 and mRS 0–2), TSA demonstrated that the required information size was achieved. The cumulative Z-curve did not cross the conventional significance threshold or the trial sequential monitoring boundary, suggesting a lack of robust evidence to demonstrate a 20% relative benefit of bridging therapy over MT alone (Figure 6). These findings further imply that additional trials are unlikely to change this conclusion, as the TSA adjusted CI was 0.89–1.23 for excellent functional outcome and 0.93–1.38 for good functional outcome. A sensitivity analysis using a 15% relative risk increase yielded comparable results, further supporting the robustness of these findings (Figure S15).

## Discussion

This study-level meta-analysis, encompassing 7 RCTs with 2884 patients, provides a comprehensive and updated assessment of the safety and efficacy of IVT prior to MT in patients with AIS due to large vessel occlusion. Our findings indicate that IVT followed by MT demonstrates comparable safety and efficacy to MT alone across excellent functional outcome, good functional outcome, successful recanalization, all-cause mortality, symptomatic ICH, and any ICH. These results are further supported by TSA, which did not confirm a 20% relative risk increase in functional outcomes.

Our meta-analysis aligns with and expands upon previous research. The IRIS (Improving Reperfusion Strategies in Ischemic Stroke) Collaborators, in their individual participant data meta-analysis, similarly found that the noninferiority of thrombectomy alone could not be definitively demonstrated when compared to the combined approach of IVT plus MT.^20^ While the IRIS meta-analysis did not demonstrate superiority of IVT plus MT, our updated analysis—incorporating additional recent trials and a larger patient population—suggests that direct MT may not be superior to bridging therapy. Moreover, TSA suggests that the likelihood of identifying a meaningful clinical benefit with further trials is low, reinforcing the conclusion of comparable efficacy between the two treatment strategies.

Previous meta-analyses have presented a mixed picture regarding the utility of bridging IVT.^6,21,22^ Some earlier studies, often including observational data or a smaller number of RCTs, suggested potential benefits of IVT + MT in terms of functional outcomes and recanalization rates, while others reported no significant differences. For instance, a meta-analysis that included both RCTs and observational studies indicated that MT + IVT patients had better functional outcomes, lower mortality, and higher rates of successful recanalization. However, the inclusion of observational studies in such analyses can introduce bias and limit the generalizability of findings.^13^ Our meta-analysis, by exclusively focusing on RCTs, provides a higher level of evidence, mitigating some of these concerns.

The observed comparable rates of successful recanalization between the two groups in our study are particularly noteworthy. While some might hypothesize that IVT could facilitate clot lysis and improve the chances of successful recanalization,^23–25^ our findings suggest that the mechanical removal of the clot remains the dominant factor in achieving reperfusion. The sensitivity analysis, where exclusion of the BRIDGE-TNK study made the finding statistically significant for successful recanalization, warrants further investigation into the specific characteristics of trials and patient populations that might influence this outcome.^26^ The significant subgroup interaction based on the thrombolytic agent used (alteplase vs. tenecteplase) also highlights the importance of considering the type of thrombolytic, as tenecteplase has shown promise in some studies due to its higher fibrin specificity and longer half-life.^27–29^ Moreover, recent randomized trials have reported comparable, and in some cases superior, efficacy of tenecteplase in terms of reperfusion and functional outcomes.^30,31^ Given its increasing adoption in clinical practice, the observed subgroup interaction emphasizes the necessity of interpreting trial outcomes in the context of the thrombolytic agent used.

Regarding safety outcomes, our meta-analysis found no significant differences in symptomatic ICH or any ICH between the IVT + MT and MT-alone groups. This is a crucial finding, as concerns about increased hemorrhagic risk have historically been a major consideration against routine IVT prior to MT.^32–34^ The absence of a statistically significant increase in hemorrhagic complications in our pooled analysis provides reassurance regarding the safety profile of bridging therapy. This aligns with some recent meta-analyses, such as the one by Du et al., which also reported no significant differences in 90-day functional outcome or mortality, but a lower rate of symptomatic ICH for direct MT.^35^ However, our study's larger sample size and exclusive focus on RCTs provide more robust evidence on this safety aspect.

The TSA conducted in our study is a valuable addition, providing insights into the sufficiency of cumulative evidence. Although the required information size was reached, TSA indicates that the current evidence remains inconclusive, and the addition of further trials is unlikely to change this conclusion. This highlights the ongoing debate and the need for continued research to refine treatment algorithms and identify specific patient subgroups that might derive differential benefits from either approach.^36^

One of the strengths of our meta-analysis is the exclusive inclusion of RCTs, which minimizes the risk of selection bias inherent in observational studies. The detailed subgroup analyses based on thrombolytic agent used provide valuable insights into potential treatment effects that might be masked in overall pooled analyses. Furthermore, beyond using conventional relative effect measures, we also calculated absolute effect sizes, conducted TSA to assess the robustness of the results, applied the GRADE framework to evaluate the certainty of the evidence, and explored a wider array of clinically meaningful outcomes.

### Clinical implications and future research directions

The observed comparable safety and efficacy profiles between IVT followed by mechanical MT and MT alone suggest that ongoing guideline discussions, particularly concerning “direct-to-angio” pathways, require a careful consideration of these findings. While some studies have indicated that direct transfer to an angiography suite may optimize workflow and improve outcomes, especially within specific time windows, our results demonstrate that the addition of IVT does not significantly enhance functional outcomes or elevate hemorrhagic risk when MT is performed.^37–39^ This implies that a “direct-to-angio” approach, which bypasses pre-MT IVT, could represent a viable strategy, particularly given the potential for increased healthcare resource utilization associated with IVT without a demonstrable superior clinical benefit when MT is readily accessible.

Future RCTs should prioritize several key areas. The significant subgroup interaction identified based on the thrombolytic agent employed (alteplase vs. tenecteplase) for successful recanalization necessitates further investigation into the optimal thrombolytic and its precise role within bridging therapy.^27,28^ Furthermore, although our TSA indicates that larger trials may not reveal a substantial difference in overall functional outcomes, subsequent RCTs could explore specific patient subpopulations that might derive differential benefits from either therapeutic approach, such as those characterized by distinct clot morphologies, collateral circulation status, or varying times to treatment. Future research may also encompass an evaluation of the cost-effectiveness of each strategy across diverse healthcare systems, accounting for the economic implications of IVT in the absence of a clear incremental clinical advantage. Ultimately, sustained efforts to optimize prehospital and in-hospital care pathways, including the “direct-to-angio” paradigm, remain important to facilitate timely reperfusion and enhance patient outcomes, irrespective of the initial thrombolytic strategy.

### Limitations

Despite these strengths, our study has certain limitations. The heterogeneity observed in some outcomes, although generally low, suggests that some variability across trials might exist due to differences in patient characteristics, treatment protocols, or geographical settings. The variability in the reporting definitions of ICH and symptomatic ICH may have contributed to the heterogeneity observed in the analysis, potentially explaining the mild heterogeneity noted for any ICH. While we conducted sensitivity analyses to explore the impact of individual studies, residual heterogeneity cannot be entirely ruled out. Additionally, the limited number of pooled studies (fewer than 10) precluded the assessment of publication bias using statistical tests, which is a common limitation in meta-analyses of a small number of trials. As our study relied on study-level data, we were unable to conduct additional subgroup analyses based on varying patients’ characteristics. Future research with a larger number of RCTs would be beneficial to address this limitation and provide even more definitive conclusions.

## Conclusion

Our study-level meta-analysis provides evidence that IVT followed by endovascular treatment offers comparable safety and efficacy to MT alone for patients with AIS due to large vessel occlusion. These findings have important implications for clinical practice, suggesting that bridging therapy remains a viable and safe option. While TSA indicates a need for further evidence to detect subtle differences, the current data support the continued use of IVT prior to MT in eligible patients. Future research should focus on identifying specific patient subgroups who may benefit most from one approach over the other, potentially through large-scale randomized trials with sufficient power to detect smaller effect sizes and explore the impact of different thrombolytic agents.
